# Structure of the host-recognition device of *Staphylococcus aureus* phage ϕ11

**DOI:** 10.1038/srep27581

**Published:** 2016-06-10

**Authors:** Cengiz Koç, Guoqing Xia, Petra Kühner, Silvia Spinelli, Alain Roussel, Christian Cambillau, Thilo Stehle

**Affiliations:** 1 Interfaculty Institute of Biochemistry, University of Tübingen, 72076 Tübingen, Germany; 2 Interfaculty Institute of Microbiology and Infection Medicine, University of Tübingen, 72076 Tübingen, Germany; 3 German Center for Infection Research (DZIF), partner site Tübingen, Germany; 4 Institute of Inflammation and Repair, Faculty of Medical and Human Sciences, University of Manchester, Manchester, United Kingdom; 5 Architecture et Fonction des Macromolécules Biologiques, Aix-Marseille Université, UMR 7257 13288 Marseille Cedex 09, France; 6 Architecture et Fonction des Macromolécules Biologiques, Centre National de la Recherche Scientifique, UMR 6098, Campus de Luminy, Case 932, 13288 Marseille Cedex 09, France; 7 Department of Pediatrics, Vanderbilt University School of Medicine, Nashville, Tennessee, USA

## Abstract

Phages play key roles in the pathogenicity and adaptation of the human pathogen *Staphylococcus aureus*. However, little is known about the molecular recognition events that mediate phage adsorption to the surface of *S. aureus*. The lysogenic siphophage ϕ11 infects *S. aureus* SA113. It was shown previously that ϕ11 requires α- or β-N-acetylglucosamine (GlcNAc) moieties on cell wall teichoic acid (WTA) for adsorption. Gp45 was identified as the receptor binding protein (RBP) involved in this process and GlcNAc residues on WTA were found to be the key component of the ϕ11 receptor. Here we report the crystal structure of the RBP of ϕ11, which assembles into a large, multidomain homotrimer. Each monomer contains a five-bladed propeller domain with a cavity that could accommodate a GlcNAc moiety. An electron microscopy reconstruction of the ϕ11 host adhesion component, the baseplate, reveals that six RBP trimers are assembled around the baseplate core. The Gp45 and baseplate structures provide insights into the overall organization and molecular recognition process of the phage ϕ11 tail. This assembly is conserved among most glycan-recognizing *Siphoviridae*, and the RBP orientation would allow host adhesion and infection without an activation step.

*Staphylococcus aureus* is a Gram-positive bacterium that causes a wide range of infections. It is a leading cause of bacteremia, infective endocarditis, as well as osteoarticular, skin and soft tissue, pleuropulmonary and device related infections^1^. Methicillin-resistant *S. aureus* (MRSA) remains a severe global problem threatening the health care system as resistance restricts treatment options to a few drugs of last resort.

All *S. aureus* genomes sequenced to date contain one or several prophages^2^,^3^. Most *S. aureus* phages can be integrated into the bacterial chromosome or exist as extra-chromosomal elements. It is known that many of these phages encode a large variety of *S. aureus* virulence or fitness factors that allow the bacterium to escape the host immune system. Among all mobile genetic elements in *S. aureus*, phages are probably most efficient in mediating horizontal gene transfer of virulence or resistance genes between strains, and across species or even genus. Therefore, phages play important roles in staphylococcal pathogenicity and adaptation of *S. aureus* to different hostile environments^2^,^3^.

The large number of staphylococcal phages sequenced to date display an extensive mosaicism in their gene structure, which is a consequence of gene shuffling among different phages that can infect staphylococcal species. The resulting mosaic gene organization is consistent with a modular evolution involving exchanges of genome modules by horizontal transfer and genetic recombination. The genetic exchanges of modules can involve single genes, protein domains, groups of genes, or even functional modules^3^.

Although phages are the most abundant and diversified biological entity on earth, each phage can only infect a limited number of bacterial strains. This specific phage-host interaction is determined, in part, by the protein recognition device located at the tip of the phage tail, which engages a receptor at the bacterial cell surface. Since bacterial cell wall polysaccharides or glycopolymers project from the cell surface and are thus easily accessible, they are the most common molecules targeted by bacteriophages^4^,^5^,^6^.

The cell wall of *S. aureus* typically contains poly-ribitol phosphate type wall teichoic acid (WTA), which is modified with D-alanine and N-acetyl-glucosamine (GlcNAc). *S. aureus* ϕ11 is often used as model to study horizontal gene transfer of virulence genes^7^. Recently it was shown that ϕ11 requires GlcNAc residues on WTA for adsorption^8^. Gp45 of ϕ11 was identified and characterized as the receptor-binding protein (RBP) of ϕ11 9. Furthermore, it was shown that ϕ11 was unable to bind to the cell wall in the absence of WTA–GlcNAc, identifying glycosylated WTA as the receptor.

Phages adopt a two-fold strategy for host adhesion. They first deploy adhesion modules on fibers or on capsid or tail that recognize the host’s cell wall glycan structures in a reversible way: this allows cell wall scanning in search for the final, specific receptor, to which they bind irreversibly^10^,^11^,^12^. This final receptor can be a protein, generally membrane embedded^12^,^13^, or a cell wall polysaccharide as observed in the case of lactococcal phages^4^,^5^,^6^. Attachment to membrane protein often requires a unique and strong attachment of the phage’s tail tip, as observed in phage T5 14,15. In contrast, the loose affinity observed between saccharides and proteins requires the presence of several attachment sites provided by a multimeric RBP carrying device, the baseplate^16^,^17^,^18^,^19^. In order to provide a foundation for understanding the initial recognition mechanism of phage ϕ11 and its receptor in the cell wall of *S. aureus*, we embarked on structural analyses of the Gp45 and baseplate of ϕ11 using X-ray crystallography and electron microscopy. The RBP structure reveals a trimer with a complex fold that can be divided, from N- to C-terminus, into a “stem”, a “platform” and a “tower”. The stem is formed by a long, severely bent triple α-helical coiled coil that features three interruptions: the first and third interruptions are both β-hairpin structures and the second is a short disordered region. A putative “hinge”-like feature is located between the second and the third interruption. The stem is followed by a “platform” of three β-propellers, and the protein terminates with a “tower” formed by a repetitive all-β domain. Platform and tower are interconnected by a fifth short triple helix buried inside of the protein on the molecule’s longest three-fold axis. An unusual iron is located at the C-terminal end of the first coiled coil and may play a role in mediating flexibility or conformational rearrangements within the helical domain. Six copies of the trimer assemble around the baseplate core. This hexameric organization is commonly observed in lactococcal *Siphoviridae*^17^,^18^, and it is compatible with host adhesion and infection in the absence of an activation step.

## Results

### Structure determination

The *gp45* gene was cloned into the pET28 vector (Novagen) for overexpression in *E. coli* as described elsewhere^9^. Briefly, the protein was expressed with an N-terminal hexa-histidine-tag, and purified by nickel-affinity chromatography and size exclusion chromatography as a trimer. Structure determination was performed with a Ta_6_Br_12_ derivative using single isomorphous replacement with anomalous scattering (SIRAS) and exploiting the non-crystallographic symmetry (NCS) present in the crystals. Initial refinement with PHENIX^20^ was followed by several runs with autoBUSTER^21^, and alternating refinement and model building^22^ cycles resulted in excellent R_free_ and R_work_ values of 21.1% and 17.5%, respectively, for the final model (Table 1). Although the map is generally of good quality, a few loops of the propeller domains have very weak electron density, explaining the persistence of a small number of outliers in the Ramachandran plot (0.5%). Of the remaining residues, 94.5% are located in regions of preferred conformation and 5% in regions that are classified as allowed.

### Overall structure of ϕ11 RBP

**Figure 1:**
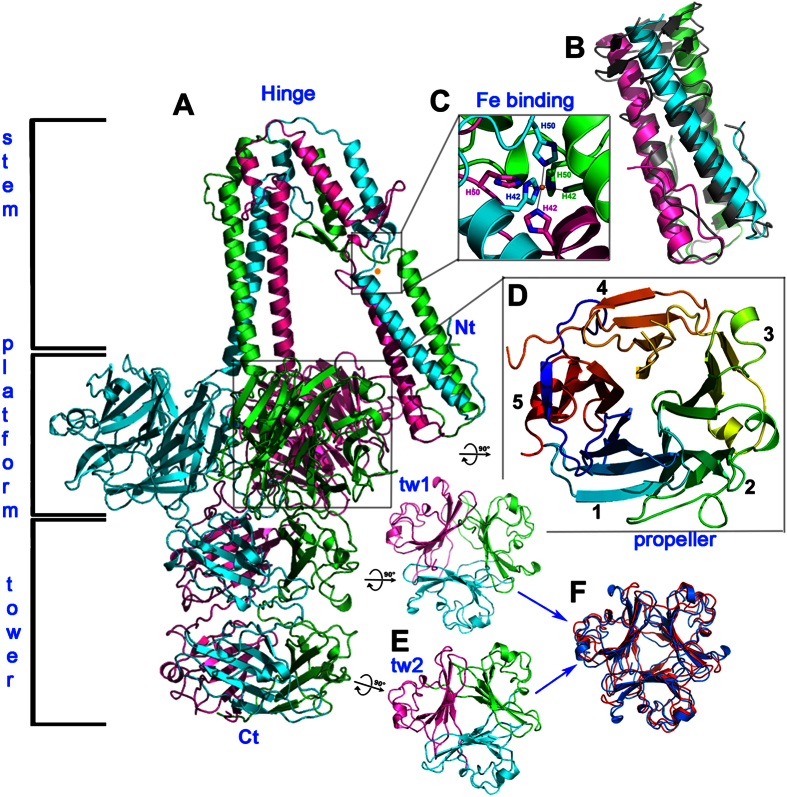
The ϕ11 RBP overall structure and domains. (**A**) The structure of the complete RBP trimer is represented as a ribbon, with monomers coloured blue, green and violet. (**B**) The trimeric N-terminus of the ϕ11 RBP has been superposed onto the 30 first amino acids of the trimeric TP901-1 RBP (r.m.s.d. of 1.8 Å). (**C**) Schematic representation of the Fe^3+^ binding motif. (**D**) The 5-bladed propeller domain rainbow coloured from dark blue (N-terminus) to red (C-terminus). The components of blade 5 are the N-terminal and C-terminal β-strands of the domain. (**E**) The two C-terminal trimeric domains, each assembling into three 4-stranded β-sheets. (**F**) Superposition of the two C-terminal domains. The r.m.s.d. between these domains after superimposition is 1.7 Å (blue and red structures).

**Figure 2:**
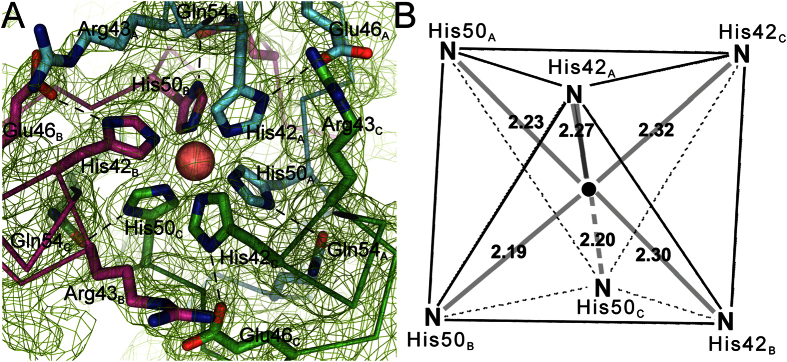
The Fe^3+^ binding motif. (**A**) Final 2F_o_-F_c_ electon density map of the Fe^3+^ binding motif contoured at 2.0 σ. The salmon-coloured sphere represents the octahedrally coordinated iron center in the histidine-rich stem region of helix-bundle α1. A second shell of residues, responsible for maintaining the active tautomeric states of the histidines, are represented with their side chains as sticks. Dashed lines indicate hydrogen-bonds and polypeptide chains are coloured blue for chain A, magenta for chain B and green for chain C. (**B**) Schematic representation of the octahedral motif, with the distances between His-Nε2 atoms from residues His 42/His 50 and the Fe^3+^ ion.

**Figure 3:**
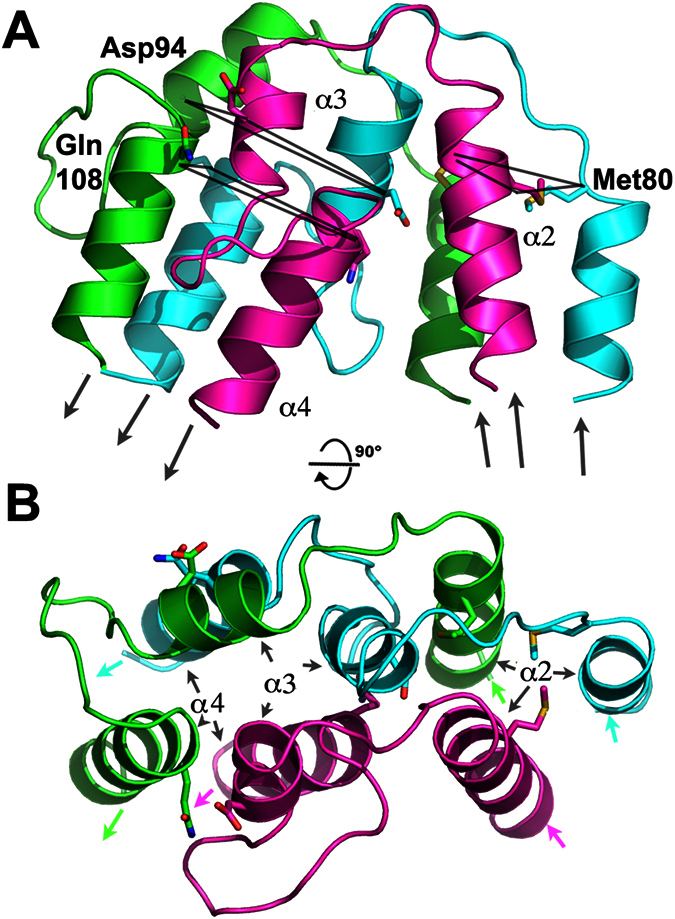
The hinge domain. (**A**) Ribbon representation of the trimeric hinge domain, encompassing amino acids Met80 to Gln108. Equivalent residues in the three monomers have been joined, and the sequence phase is conserved at this position. The direction of each chain is indicated by arrows. (**B**) 90° rotated view.

The RBP of ϕ11 assembles into an elongated homotrimer, with overall dimensions of approximately 160 × 120 × 100 Å (Fig. 1A). The structure can be divided into an N-terminal “stem” region that forms a triple-helical bundle (Figs 1B and S1), a central “platform” region composed of three β-propeller domains (Figs 1D and S1) and a C-terminal “tower” region (Figs 1E and S1). Overall, the stem contains three non-helical interruptions. The first of these occurs between residues 46 and 67 and contains a bound iron as well as a β-hairpin that faces away from the bundle axis (Figs 1C and 2). The second and the third interruptions are located between residues 81 and 107, which introduce a sharp kink into the stem (the “hinge”) and thus break the shared three-fold symmetry of α1 and α2 (Figs 1 and 3). Helix α3 (residues 88–97), a short triple helical coil located in the hinge, has an independent rotation axis not aligned to the remainders of the molecule (Fig. 3). This helical bundle is followed by five-bladed β-propeller modules of the platform, which encompass residues 142–439 and form the midsection of the protein (Fig. 1). This “platform” is linked via a short helix (residues 425–432) to the C-terminal “tower”. The latter contains two structurally similar domains (residues 440–541 and 542–636), which are each formed by three five-stranded anti-parallel β-sheets, one from each monomer, that are covered on their surface-exposed side by loops and one short α-helix each (Fig. 1).

Each ϕ11 RBP monomer forms extensive contacts with the two others monomers in the trimer. For each contact, 2 × 5,800 Å^2^ (11,600 Å^2^) are buried in the interaction as calculated by PISA^23^. This results in a total buried surface of ~35,000 Å^2^ for the trimer. Most of the buried surface area is concentrated in the stem and C-terminal regions, while the propeller domains engage in few intermolecular contacts (Fig. S2).

### The stem structure

The stem comprises three separate triple-helical bundles, which are composed of helices α1, α2 and α3/α4, respectively (Fig. 1B). The helices pack tightly together in each of the bundles, and almost every residue of each monomer is in contact with a residue of one of the two other monomers (Fig. S2) through central hydrophobic contacts or lateral hydrogen or ionic bonds. The trimeric ensemble comprising the extended N-terminus and helix α1 can be superposed onto the 30 first residues of phage TP901-1 RBP (PDB code 3U6X) with an r.m.s.d. value of 1.8 Å for 90 Cα atoms (Fig. 1B). The remaining helical bundles most closely resemble those found in phage TP901-1 Baseplate protein Upper (BppU) and other viral trimeric helix bundles^18^,^19^.

A strong electron density feature suggesting the presence of a metal ion was observed at the junction between the first two bundles. Using an RBP crystal and extended X-ray absorption fine structure (EXAFS) spectroscopy, the identity of this ion was determined to be iron, which probably exists in its oxidized form Fe^3+^ (analyzed at SOLEIL beamline PX1) (See Fig. S3). The Fe^3+^ ion is positioned along the 3-fold axis of the first helix bundle and coordinated by the side chains of His42 and His50 from each of the three monomers (Fig. 1C). This gives rise to a near-perfect octahedral coordination, in which the HisNε_2_-Fe distances range from 2.19 Å to 2.32 Å. His42_X_ is in close hydrogen-bond distance to an acid/base pair, which forms a second shell around the His-Fe-octahedron. The side-chain functional groups of Glu46_X_ and Arg43_Z_ allow for an arrangement in which the deprotonated glutamate-carboxylate is oriented to His42_X_-Nε_1_ (2.66–2.81 Å), forcing His42_X_-Nε_2_ to point towards the Fe-center. A similar tautomerization effect might occur for the diametrically opposed His50_Z_, as it is in close distance to Gln54_Z_ (3.24–3.27 Å), forcing His50_Z_-Nε_2_ to coordinate to Fe^3+^, resulting in an intertwined chelate-complex comprised of all three protein chains (see Fig. 2). It is noteworthy that the His42-Nε_2_-Fe^3+^ distances are comparable (2.295 ± 0.025 Å) but significantly longer than the His50-Nε_2_-Fe^3+^ distances (2.21 ± 0.02 Å). Such a Fe^3+^ binding geometry has previously been observed in the membrane-piercing spike proteins of phages P2 (PDB code 3QR7) and ϕ92 (PDB code 3PQH)^24^, as well as in the receptor-binding domain of the long tail fiber of phage T4 (PDB code 2XGF)^25^. In the three reported cases, the Nε_2_-Fe distances were 2.20 ± 0.01, 2.23 ± 0.01 and 2.31 ± 0.06, respectively, compared to an average of 2.25 ± 0.07 Å for the distances observed in ϕ11 RBP. The distance values in ϕ11 RBP are also close to the averages of those reported for N-Fe^3+^ bonds in average-resolution and high-resolution protein structures deposited in the PDB, 2.25 ± 0.15 Å and 2.16 ± 0.13 Å, respectively^26^. However, all of these values are larger than those observed for distances between a heme Fe^3+^ and the Nε_2_ of histidines coordinating it axially in myoglobin (2.00–2.11 Å)^27^. Indeed, iron ions often absorb in visible light wavelength ranges, giving rise to a red color for hemes and a brownish color for Fe-S clusters. However, the ϕ11 RBP and the related phage proteins discussed above are all colourless in solution^24^. It is worth noting that the Fe^3+^ binding regions in the phage P2, ϕ92 and T4 spike structures involve histidines within a His-X-His motif at the apex of an intertwined triple β-helix. It has been proposed that this ion binding structure might strengthen the puncturing device of phages that pierce the cell wall^24^. In ϕ11 RBP, the His42-X_7_-His50 motif lies at a junction between two helical bundles. We therefore suggest that it serves a different role, perhaps by helping to stabilize the bundles that undergo a sharp turn at the hinge. If the Fe lock would not be in place, the structure of the α1/α2 segment would likely not be maintained as a rigid unit.

While the first and second helical bundles are collinear, the hinge introduces a sharp angle of ~30° between the second and the third bundle. This angle is the smallest possible since the second and third bundles are in contact at positions 61–64 and 116–121, while the first bundle contacts the propeller domain at position 219 (Figs 1 and S1). The hinge geometry is such that the sequences of the three helices of bundles two and three remain in phase (Fig. 3). The second bundle terminates with Met80, and the following sequences in the three monomers adopt a coil structure that abuts helices α3 where the three sequences are already in phase (see Asp94, Fig. 3). Helices α3 are followed by extended hairpin-structures and helices α4 forming the final bundle.

### The five-bladed propeller platform and the two C-terminal tower domains

The C-terminal end of the third helical bundle abuts the three five-bladed propeller domains that form the platform of ϕ11 RBP (Fig. 1D). The three propellers are all equidistant to each other and to the molecule’s main NCS-axis. This whole platform domain occupies a space that is ~100 Å wide and ~40 Å thick. Contacts between the three propeller domains are sparse, as each interface between two propeller domains buries a surface area of only 457 Å^2^ from solvent, and much of this surface is buried due to a helix-helix contact at the center of the trimer axis (Fig. S2). The plane of the propeller is not perpendicular to the 3-fold axis, but is tilted upwards (as represented in Fig. 1) by an angle of ~30°. This tilt improves access to the lower face of the propeller, and this might be linked to the function of RBP in interacting with ligands (see below).

As in other propeller structures, sets of four anti-parallel β-strands form each blade, and the N-terminal β-strand closes the fold by forming the final blade (blade 5) with the three C-terminal β-strands of the domain (Fig. 1D). A DALI search^28^ with the ϕ11 RBP propeller returned many significant hits above a Z-score of 15, and with r.m.s.d. values ranging from 3.1 to 4.0 Å. Most of the identified proteins are enzymes that mediate the degradation of carbohydrates. The highest score (Z = 16.1, r.m.s.d. = 3.1 Å), however, was obtained for the enzyme glutamine cyclotransferase from *Zymomonas mobilis* (PDB code 3NOL)^29^. To our knowledge, only two other examples of β-propellers in putative RBPs have been reported. For one, a distorted five-bladed propeller has been identified as the head domain of the RBP-P2 protein of phage PRD1, a *Tectiviridae* member infecting Gram-negative bacteria (PDB code 1N7U)^30^. The second example is the C-terminal domain gp131C of the *Pseudomonas* myophage PhiKZ, forming a seven-bladed β-propeller domain (PDB code 4GBF)^31^, and its position at the periphery of the baseplate has led to speculation that the propeller might act as the receptor-binding domain or as a cell-degrading enzyme. Neither of these hypotheses, however, have been confirmed experimentally.

**Figure 4:**
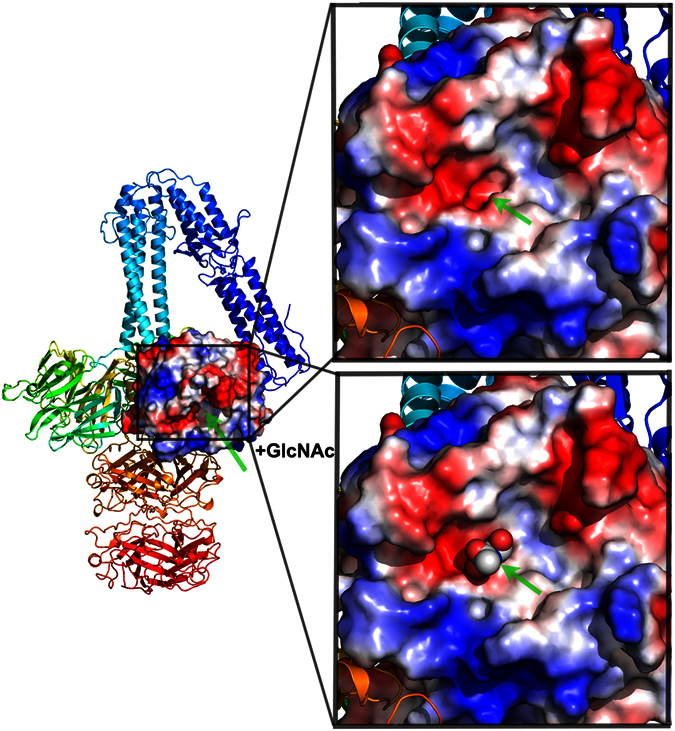
The platform domain. The complete structure as a ribbon view with the surface of the five-bladed β-propeller domain coloured according to its electrostatic surface potential. **Inset above**: close-up view of the domain evidencing a deep cavity at its center. **Inset below**: model of a GlcNAc molecule (the main receptor’s component) docked into the cavity.

When the *Zymomonas mobilis* glutamine cyclotransferase structure^29^ was superimposed onto the RBP propeller domain, the active site of the enzyme overlaid a deep crevice located within the RBP lower face (Fig. 4). Modelling indicates that a cavity in this crevice has the correct size to accommodate a GlcNAc molecule, the cell wall teichoic acid (WTA) component specifically recognized by phage ϕ11 9. Three water molecules occupy this cavity (Fig. S4A), which can be nicely replaced with hydroxyl groups of a modelled GlcNAc molecule^8^. The cavity is lined with polar residues (Gln165, Thr211, Gln330), which could serve to establish hydrogen bonds with the modelled GlcNAc molecule (Fig. S4B). Apolar residues Met164 and Met329 complete the walls of the cavity.

The two C-terminal tower domains form a structure of dimensions 60 × 50 × 50 Å. These two domains are very similar in structure, which is confirmed by a superposition that yields a low r.m.s.d. value of 1.7 Å for their Cα atoms (Fig. 1E,F). A DALI search^28^ performed with these domains returned only lower Z-scores, with the highest of these (Z = 6.1; r.m.s.d. = 3.2Å) for uracil-DNA glycosylase inhibitor, a small all-β monomeric protein (PDB code 2UGI). The two structures essentially share the same anti-parallel β-sheet but differ in their oligomeric state and their surrounding structural features, and the identity (9%) is not high enough to assign possible functions to the C-terminal domains.

### Negative staining electron microscopy structure of the ϕ11 baseplate

**Figure 5:**
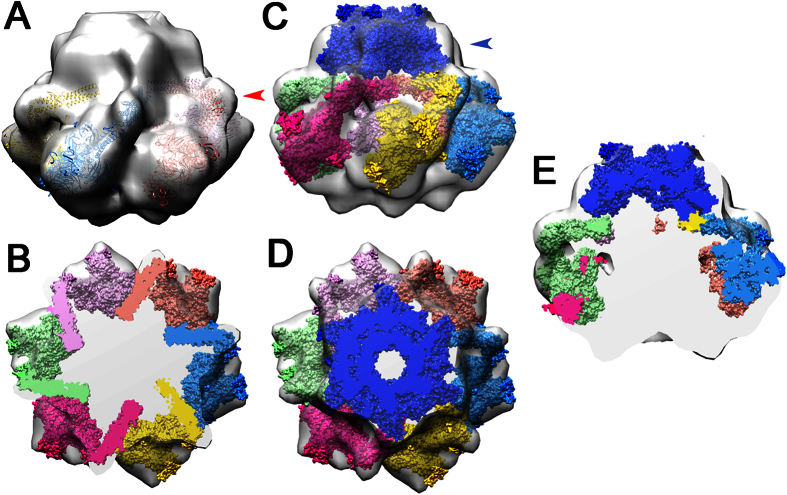
Electron microscopy negative-staining map of the RBP baseplate. (**A**) The electron microscopy map is displayed with the six RBPs, represented as ribbons, fitted manually into the map using Chimera^71^. The red arrow indicates the hinge that was modified to provide a good fit. (**B**) A section of the baseplate with the six RBPs cut as shown in A. (**C**) View of Dit and the six RBPs fitted in the baseplate. The blue arrow indicates the Dit structure. (**D**) A section of the baseplate with Dit cut as shown in C. (**E**) Vertical slicing of the baseplate showing the cut volumes of Dit and the RBPs as well as the empty volume putatively occupied by the Tal and Gp54 proteins.

To define the topology of the ϕ11 baseplate and allow location of the RBP, we conducted electron microscopy analysis using negative staining of the virion. This approach has been successful in other cases^17^,^18^,^32^,^33^. We collected 512 images of the phage, and boxed 778 baseplate particles (see experimental procedures section). The final map has a resolution of 23 Å (determined using the 0.5 FSC criterion) and allowed us to unambiguously place six ϕ11 RBP trimers (Fig. 5A,B). To optimize this fit, we modified the hinge angle between the second and the third helix bundles from a value of ~30° to ~90°. The correlation is 0.845 with 95.5% of the atoms inside the map, calculated for a RBP orientation fit with the tower domain inclined towards the bottom of the baseplate. Compared to this, an orientation in which the tower domain would be “heads up”, reminiscent of the resting state of p2 baseplate^17^, only resulted in a correlation of 0.826 with 74.9% of the atoms inside the map. However, it has to be taken into account that the “heads-down” conformation is not a completely non-flexible state. The angle of the stem might in fact vary from the minimum observed in the X-ray structure to larger values when the phage scans the host’s surface by moving the RBPs around the calculated average position for adhesion to the specific receptor. Such movements have been observed for several phages, such as phage T7 10,11.

The remainder of the RBP structure was left unaltered, and the modified trimers fit well in a peripheral region of the map that could accommodate the triangular shape of the platform domain’s platform. In order to explain the remaining density of the baseplate, we performed HHPRED^34^ analyses of ϕ11 proteins Gp43, Gp44 and Gp54, which are the most likely candidates for baseplate components^9^. This analysis revealed similarities with components of the lactococcal phage TP901-1 baseplate^9^, suggesting that the central part of the ϕ11 baseplate is organized similarly to that of TP901-1 18. Based on this analysis, Gp43 is predicted to exist as a hexamer and form the distal tail protein (Dit) ring and Gp44 as a trimer forming the tail-associated lysin (Tal) N-terminus and extension, while Gp54 N-terminus (the functional equivalent of BppU N-terminus) may form a second ring. Furthermore, the N-terminal segment and the first helical bundle of ϕ11 RBP are structurally homologous to the N-terminal part of the phage TP901-1 RBP trimer, a structural domain that anchors the RBP into the BppU C-terminus^18^. We therefore also attempted to fit the phage TP901-1 Dit hexamer together with the BppU N-terminus (amino acids 1–160) into our electron density map^18^. The ring of the Dit had appropriate dimensions to fit the map above the RBPs (Fig. 5C,D). In contrast, the structure equivalent to BppU could not be fitted unequivocally as the internal density is not defined sufficiently. A large volume of the EM map remains to account for the Gp54 and for the Tal (Fig. 5E). When attaching the trimeric Tal N-terminal domain below the Dit hexamer, the three carbohydrate binding modules (2WAO) identified by HHpred project in the direction of the tail tip. These three bulky modules should fill the electron density map in between the six RBP trimers.

## Discussion

We have solved the crystal structure of ϕ11 RBP and located this protein in the tail spike of the assembled phage using electron microscopy. Our analysis defines the domain organization of RBP, which can be divided into a stem region, a platform domain and a tower-like C-terminal structure composed of two nearly identical domains. Interestingly, the stem displays a severely bent, hook-like conformation that may undergo a conformational change as the protein can only be fitted into the electron density of the tail spike in a less bent arrangement. Unexpectedly, the stem also contains a bound iron. The function of this iron is unknown as its location differs from irons found in other spike proteins. The platform region is formed by three propeller domains and likely harbours the binding site for the substrate GlcNAc. Although soaking and cocrystallization experiments with GlcNAc were not successful, modelling suggests a reasonable location for the GlcNAc binding site in the platform region. Of note, the propeller fold was identified in the endosialidases of several phages. These enzymes cleave polysialic acid at the surface of their host in order to obtain access to the capsular cell wall. For example, coliphages K1F and phi92 possess such endosialidases, which also exhibit trimeric propeller domains attached to a stem^35^,^36^.

Comparison with the phage TP901-1 tail spike assembly allows us to also assign a putative location of the Gp43 and Gp54 proteins of ϕ11. Gp43 likely forms the hexameric Dit, while the N-terminus of Gp54 resembles the first 160 residues of TP901-1 BppU^18^. The remainder of the electron density is likely occupied by the rest of the large Gp54 and by Gp44, the Tal protein. Interestingly, the N-terminal folds of Dit and Tal are found in a wide range of phages^37^, including *Myoviridae* infecting Gram-negative bacteria (T4 or Mu^38^,^39^), *Siphoviridae* from Gram-negative (T5 40) or Gram-positive (SPP1^41^,^42^) bacteria, lactococcal phages^17^,^33^,^43^,^44^, or even mycobacteria (Araucaria^45^). It is worth noting that the Tal protein is also found in the type VI secretion system machinery^46^. This observation suggests that the block formed by Dit and Tal could have been conserved through evolution, a phenomenon shared by other components such as the capsids MCP^47^,^48^, the connector^49^, as well as the tail MTP^50^. Only the periphery of Dit (its C-terminal domain) and the Tal extension (e.g. a C-terminal fiber) could have been adapted to specific phage infection-style requirements^51^. In contrast, ϕ11 Gp45, the RBP, does not exhibit analogy with other phage RBPs, in particular with those from lactococcal phages that also bind to saccharidic receptors. Lactococcal phages p2 52, TP901-1 53, Tuc2009 19, bIL170 54 and 1358 4 all possess a trimeric receptor recognition head sharing a *bona fide* or a modified jelly-roll motif. The rest of their RBPs share common motifs in the neck or in the N-terminal domain (or stem). In Gp45, only the first 30 amino-acids of the stem resemble those of phages TP901-1 or Tuc2009.

Although the phage TP901-1 BppU protein does not seem to share such an extensive evolution coverage, the presence of a large part of it either in the RBP (Gp45) or in the subsequent protein (Gp54) was quite surprising. This finding suggests that phages might not capture only widespread elements in the protein domains repertoire, but also less diffused components, even between remote phages with different hosts. In the present case, the role of Gp54 is not documented and difficult to predict. We think it likely that in phage ϕ11 Gp54, a large C-terminal domain might also accommodate the RBP N-terminus, but with different structural features compared to TP901-1 BppU, because of its much increased size. The electron microscopy low-resolution structure gives hints of the putative receptor binding sites, located below the five-bladed propeller domain. This arrangement allows for a correct orientation to capture the GlcNAc of the cell wall teichoic acids. Although, a well-defined cavity in the size of a monosaccharide exists, a much larger crevice surrounds this cavity, suggesting that other WTA components might complement the interaction. However, further structural data are necessary to develop this hypothesis.

## Experimental Procedures

### Overexpression, purification and crystallization of Gp45

Gp45 was produced and purified as described elsewhere^9^. Briefly, after induction with IPTG the protein was purified to homogeneity using nickel-affinity chromatography and size exclusion chromatography. The purified protein carries a hexa-histidine tag at its N-terminus. Two similar crystallization solutions (0.1 M bicine/Trizma base pH 8.5, 10% w/v PEG 8000, 20% v/v ethylene glycol, 0.12 M monosaccharide-mix^55^) yielded initial crystals (50 × 20 × 5 μm) of triangular shape that grew to bouquets at 16 °C over 1 week. Reproduction of the crystals in 5 μL hanging drops lead to bigger crystals (500 × 200 × 50 μm) that were used for X-ray structural analysis.

### Phasing, construction, refinement

Data for native and derivative crystals were collected at the Swiss Light Source (SLS) on beamline X06DA (PXIII) using a PILATUS 2M hybrid pixel detector. For the determination of peak, inflection, high-remote and low-remote wavelengths from fluorescence spectra, the program CHOOCH was adjusted to the absorption edges of Ta-L-II (1.11325 Å peak) and Ta-L-III (1.25476 Å peak). Data were processed with the XDS package^56^. The crystals belong to spacegroup P1 and have unit-cell dimensions a = 87.06 Å, b = 89.01 Å, c = 93.26 Å, α =  93.0°, β = 105.2° and γ = 117.6°. A Ta_6_Br_12_ derivative (a = 87.65 Å, b = 89.60 Å, c = 93.73 Å, α =  92.7°, β = 105.7° and γ = 117.9°) was prepared by soaking native crystals in crystallization solution + 2mM Ta_6_Br_12_ for up to 2 weeks before backsoaking in crystallization condition and vitrification in liquid nitrogen. Anomalous data were processed according to MAD, SAD, MIRAS and SIRAS protocols using SHARP/autoSHARP^57^. Initial heavy atom coordinates and B-factors found with SHELXDE^58^ were reedited with the SHARP-module Sushi and were refined until electron density maps showed good contrast. The outcomes of the various phasing protocols were compared, and the map derived from the SIRAS protocol was selected for further improvement.

A threefold NCS was elicited from the self-rotation function via polarrfn (ccp4 59), giving a strong signal for rotation in reciprocal space for eulerian angles (α = 357.4, β = 63.6, γ = 110.6) corresponding to polar angles (o = 37.4, ϕ = 33.4, κ = 120.1). Using this self-rotation solution, GETAX^60^ was able to find a set of translation vectors for the asymmetric unit in real space.

Due to the size of the multi-domain protein, it was split in various parts for further processing: two for the stem (before and after the ‘hinge’), the platform domain and the C-terminal tower domain. Molecular masks^61^ were created for each part and by generating correlation maps of them separately, a set of NCS operators could be assigned to each of them. The NCS-matrices were refined with IMP and averaging with AVE^62^,^63^,^64^ converged the respective domains to about 80–90% of correlation. Each subdomain was integrated with respective NCS-matrices into a DM script for a combined density modification^65^,^66^. Starting at 5.8 Å, 80 cycles of consecutive solvent flattening, NCS-averaging, histogram matching and phase extension to a final resolution of 2.9 Å resulted in an interpretable map, which was clearly distinguishable from the unbiased calculated map. Initial refinement was carried out with REFMAC5^59^,^67^ and PHENIX^20^, and after each step model building was done in COOT^22^. The final rounds of refinement were performed with autoBUSTER^21^, leading to Rfree/Rwork values of 21.1 and 17.5% (Table 1). A portion of the electron density map is shown in Fig. 3. Structural images were generated using pymol^68^.

### Negative staining electron microscopy

Phages were purified as previously described^9^. Purified ϕ11 phage (5 μL, 10^9^ pfu) was applied to glow-discharged carbon-coated grids and left to adsorb for one min. Sample excess was blotted off and the grids were stained with 10 μL of 1% uranyl acetate for 30 sec. Micrographs (512) were recorded on a 2Kx 2K FEI Eagle CCD camera using a Tecnai Spirit electron microscope operated at 120 kV and a magnification of 48,500 (resulting in a pixel size of 4.83 Å/pixel) (Fig. S5A). The three-dimensional reconstruction was produced using a single particle procedure and the XMIPP software package^69^. Particles defined around the baseplate (778) were manually picked and subjected to maximum likelihood (ML) classification and alignment implemented in Xmipp^70^ imposing a 6-fold symmetry. The initial volume was determined using a random sample consensus (RANSAC) approach^69^ with 5 2D classes. The resolution of the final volume was estimated at 23 Å using the Fourier Shell Correlation (FSC) 0.50 criterion (Fig. S5B).

### Molecular fitting and structure visualization

Molecular graphics and analyses were performed with the UCSF Chimera package (Resource for Biocomputing, Visualization, and Informatics at UC-San Francisco)^71^. The model/EM map fitting was performed by the option “fit in map” of the “volume” register. The Dit fitting resulted in a correlation coefficient of 0.85 with 94% of the atoms in the map volume. The correlation coefficient calculated for six RBPs, with an orientation of the tower domains inclined towards the bottom of the baseplate, is 0.845 with 95.5% of the atoms inside the map.

#### Data deposition

X-ray structures and structure factors have been deposited with the Protein Data Bank (PDB, www.rcsb.org) under accession code 5EFV. The EM map of the baseplate reconstruction has been deposited at the Electron Microscopy Data Bank (EMDB, emdatabank.org).

## Additional Information

**How to cite this article**: Koç, C. *et al*. Structure of the host-recognition device of *Staphylococcus aureus* phage ϕ11. *Sci. Rep.* **6**, 27581; doi: 10.1038/srep27581 (2016).

## Supplementary Material

Supplementary Information

## Figures and Tables

**Table 1:** X-ray data collection, phasing and refinement statistics of Gp45.

	Derivative Ta6Br12	Native
Data collection		
Space group	P1	P1
Cell dimensions		
a, b, c (Å)	87.65, 89.60, 93.73	87.06, 89.08, 93.3
α, β, γ (°)	92.7, 105.7, 117.9	93.0, 105.2, 117.6
Resolution (Å)	50–3.30 (3.37–3.30)	44.4–2.20 (2.38–2.20)
R_meas_	12.5 (100.4)	6.6 (57.5)
CC (1/2) (%)	99.9 (87.1)	99.8 (83.5)
I/σI	20.4 (2.9)	12.6 (2.4)
Completeness (%)	99.3 (95.0)	97.1 (97.1)
Redundancy	13.7 (12.0)	3.2 (3.2)
Phasing		
Sites	12	
Anomlaous phasing power	1.4 (0.143)	
Figure of merit - acentric	0.26 (0.05)	
Refinement		
Resolution (Å)		44.4–2.20 (2.26–2.20)
No. reflections		116,564 (8497)
R_work_/R_free_ (%)		17.5/21.1 (21.7/25.1)
No. atoms		
Protein		15324
Ligand/ion		1
Water		1446
B-factors		
Protein		51.1
Ligand/ion		33.6
Water		54.2
R.m.s deviations		
Bond lengths (Å)		0.010
Bond angles (°)		1.12
