# Supplementary material for: Structure of the host-recognition device of Staphylococcus aureus phage ϕ11
Source: Sci Rep. 2016 Jun 10;6:27581. doi: 10.1038/srep27581 (PMC4901313; doi:10.1038/srep27581)
Supplement: Supplementary Information [file srep27581-s1.doc]

**Structure of the host-recognition device of *Staphylococcus aureus* phage φ11**

**Supplementary information**

Cengiz Koç^1^, Guoqing Xia^2, 3, 4^, Petra Kühner^2^, Silvia Spinelli^5, 6^, Alain Roussel^5, 6^, Christian Cambillau^5, 6^ * and Thilo Stehle^1, 3, 7^ *

1. Interfaculty Institute of Biochemistry, University of Tübingen, 72076 Tübingen, Germany
2. Interfaculty Institute of Microbiology and Infection Medicine, University of Tübingen, 72076 Tübingen, Germany
3. German Center for Infection Research (DZIF), partner site Tübingen, Germany
4. Institute of Inflammation & Repair, Faculty of Medical and Human Sciences, University of Manchester, Manchester, United Kingdom
5. Architecture et Fonction des Macromolécules Biologiques, UMR 7257 CNRS,13288 Marseille Cedex 09, France.
6. Architecture et Fonction des Macromolécules Biologiques, Centre National de la Recherche Scientifique, UMR 6098, Campus de Luminy, Case 932, 13288 Marseille Cedex 09, France
7. Department of Pediatrics, Vanderbilt University School of Medicine, Nashville, Tennessee, USA

*Correspondence to:

Thilo Stehle, E-mail: [thilo.stehle@uni-tuebingen.de](mailto:thilo.stehle@uni-tuebingen.de)

or Christian Cambillau, E-mail: [ccambillau@gmail.com](mailto:ccambillau@gmail.com)

**
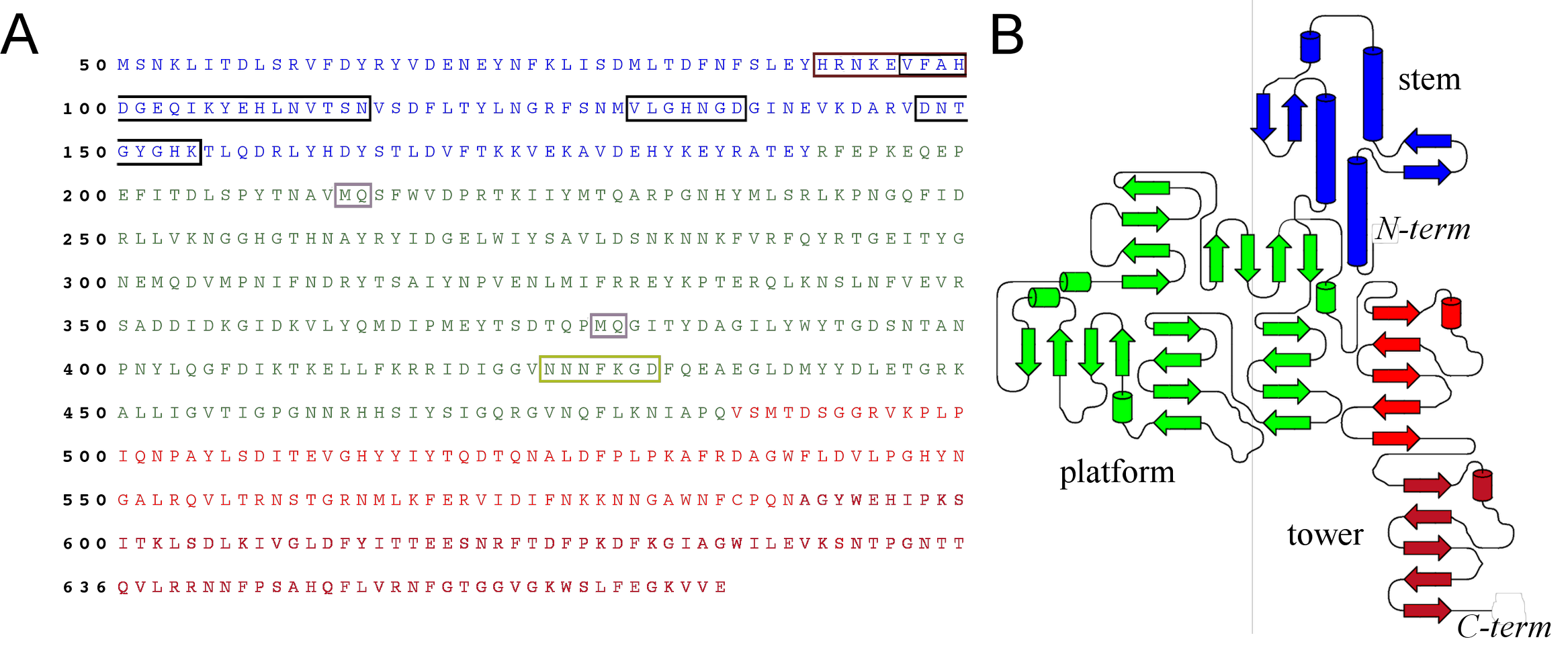
**

**Supplementary Figure S1. Primary and secondary structure of one Gp45 protomer. A/** Colour-coded sequence of Gp45 monomer: “Stem” in blue, “platform” in green, “tower1” in light-red and “tower2” in dark-red. Highlighted in coloured boxes are special features: the iron-binding-site in dark red, the interruptions in the stem in black, the putative GlcNAc binding motif in pink and the disordered loop in yellow. **B/** Topology of one Gp45 protomer partitioned in a colour-code according to Figure S1A.

**
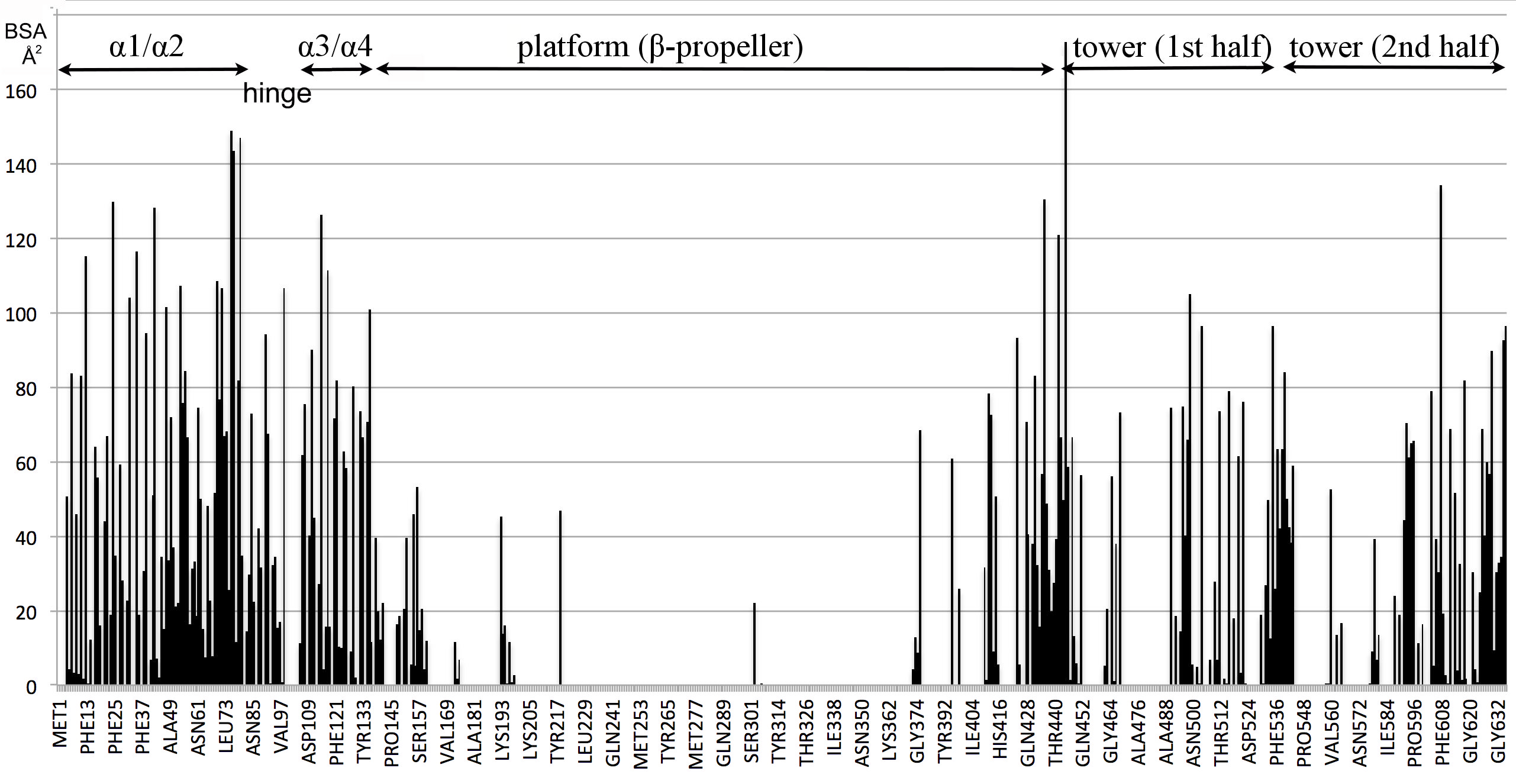
**

**Supplementary Figure S2. Buried surface area along the Gp45 chain.** The water accessible surface area of one monomer, buried by the two other monomers, has been calculated by PISA [^12^](#_ENREF_12) for each residue. Note the extensive interaction of the trimers along the stem (amino acids 1-140), while practically no interaction is observed between the β-propeller domains.

**
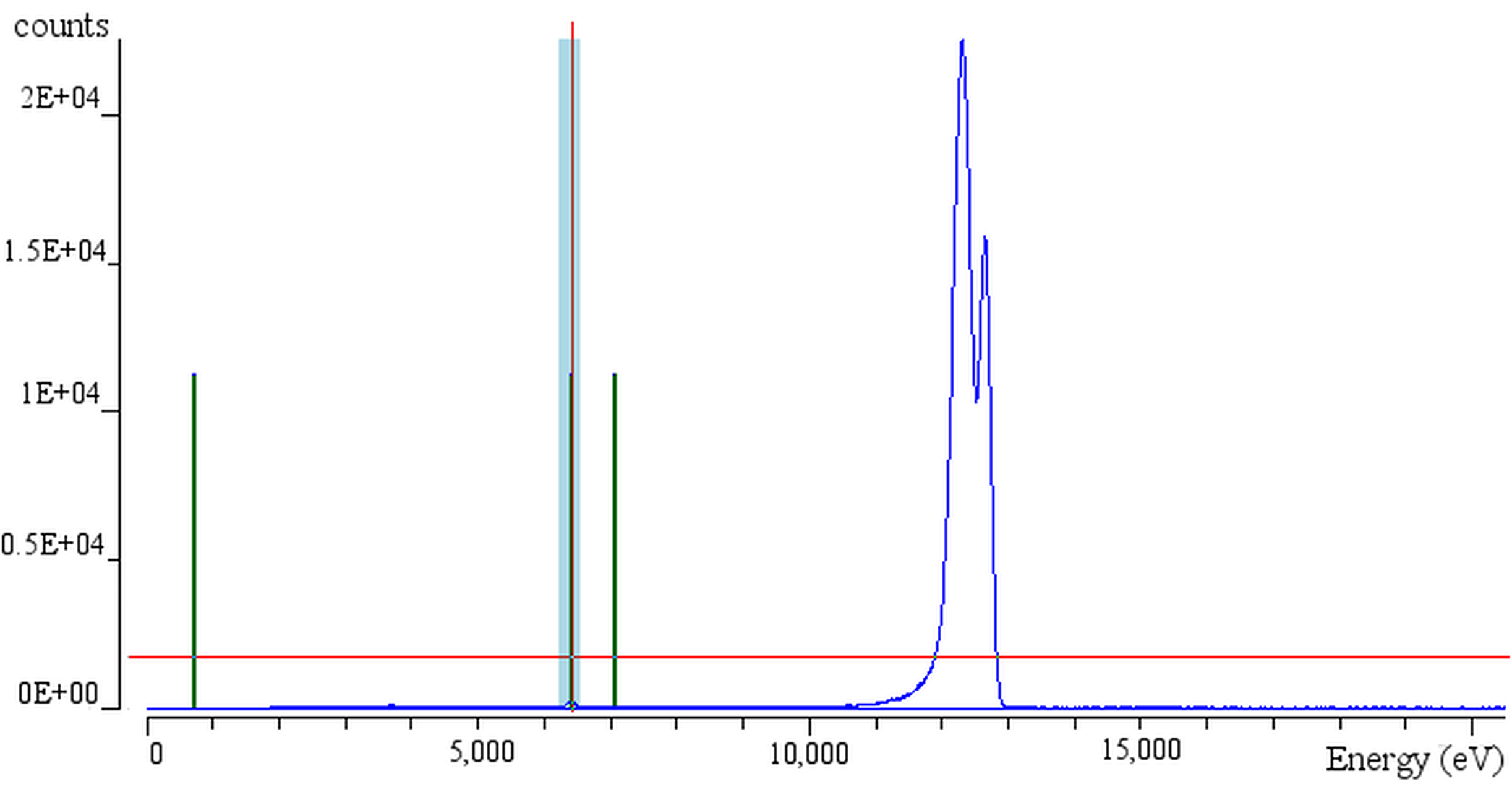
**

**Supplementary Figure S3. EXAFS spectrum of φ11 RBP crystal.** X-ray fluorescence spectra obtained upon illumination of a φ11 RBP crystal by 12.65 keV incident X-rays. The only detectable fluorescent line emission corresponds to the iron K-α energy at 6.40 keV (highlighted in light blue). The large peak on the right corresponds to the diffused scattering of incident X-rays.


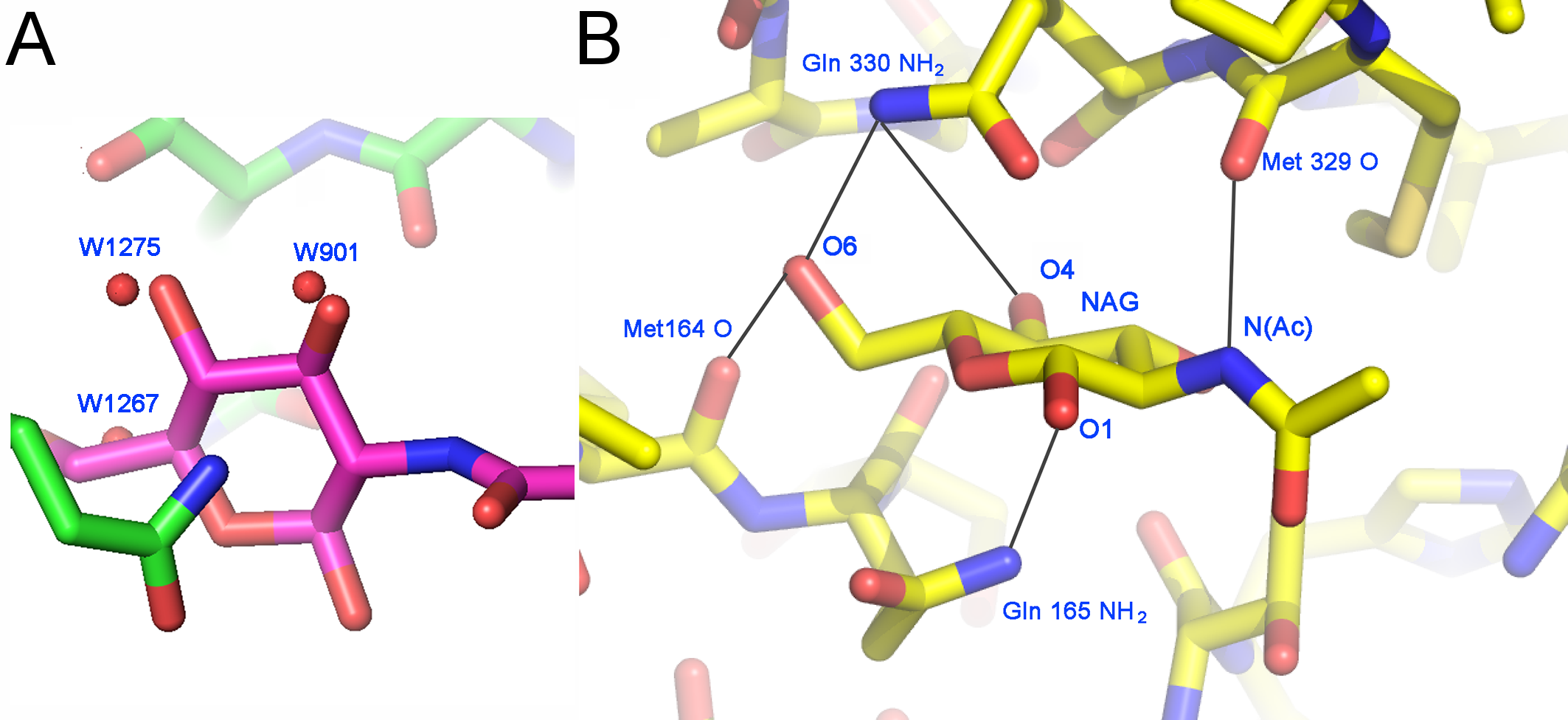


**Supplementary Figure S4. Putative GlcNAc binding-site. A/** Hydroxl groups O3, O4 and O6 of a GlcNAc substrate can be overlayed with W901, W1275 and W1267, respectively, defining a specific binding mode for a glucose residue. **B/** The overlay shown in A facilitates the modelling of GlcNAc, a major moiety of staphylococcal peptidoglycan, in a preformed cavity. The peptide-oxygen of M164 and the sidechain of Q165 of blade 2 are in appropriate proximity of O6 and O1, respectively, allowing for hydrogen bonds. The second half of GlcNAc is accommodated by M329 and Q330 of blade 5, allowing for hydrogen bridges to the nitrogen of the N-acetyl moiety, O4 and O6, respectively. This arrangement keeps GlcNAc stably in position.

**
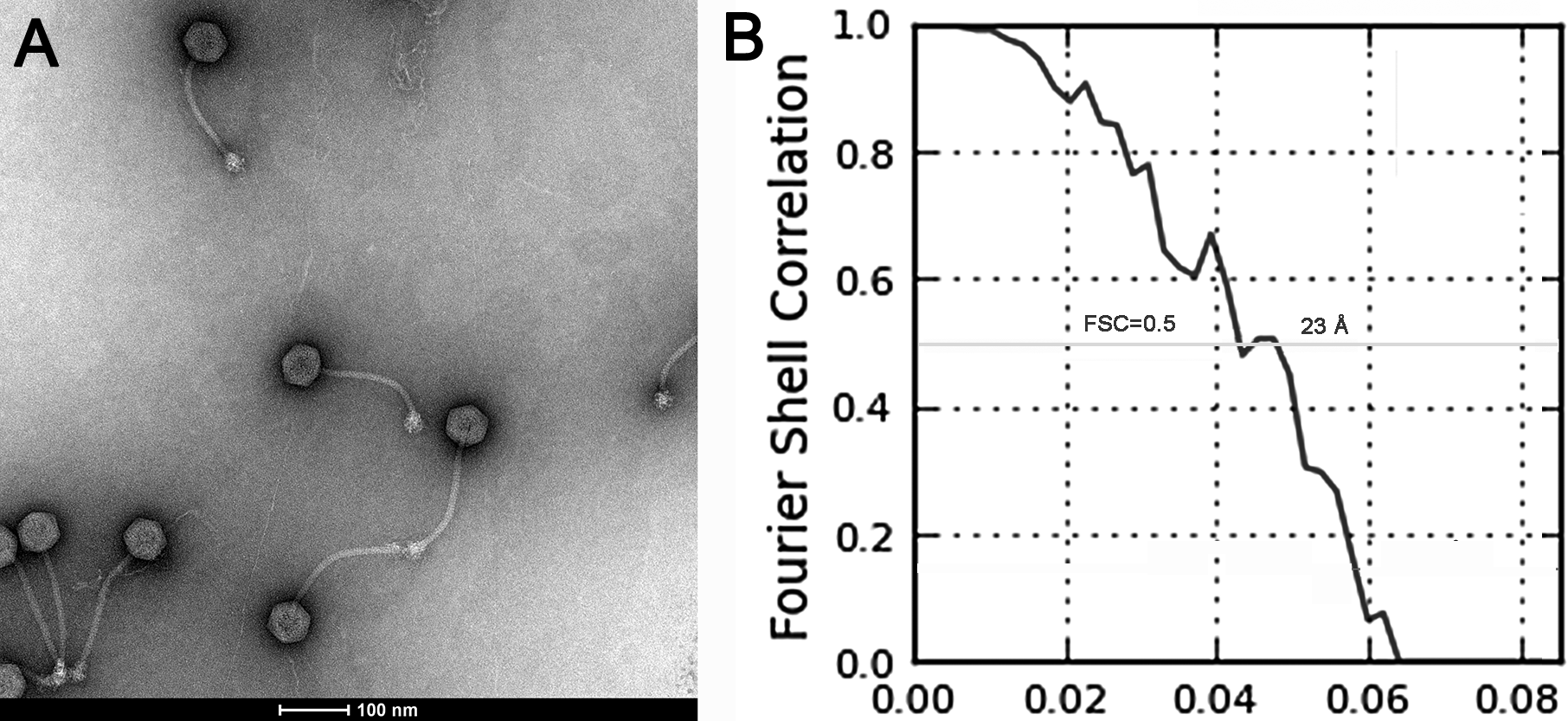
**

**Supplementary Figure S5. Electron microscopy of phage φ11. A/** An EM picture of the whole phage φ11. **B/** The Fourier shell correlation curve plotted against 1/resolution. The FSC 0.5 cut-off yields a resolution of 23 Å.
